# Comparing the Efficiency of Single-Locus Species Delimitation Methods within Trochoidea (Gastropoda: Vetigastropoda)

**DOI:** 10.3390/genes13122273

**Published:** 2022-12-02

**Authors:** Bingyu Guo, Lingfeng Kong

**Affiliations:** 1 Key Laboratory of Mariculture, Ministry of Education, Ocean University of China, Qingdao 266003, China; 2 Sanya Oceanographic Institution, Ocean University of China, Sanya 572000, China; 3 Laboratory for Marine Fisheries Science and Food Production Processes, Qingdao National Laboratory for Marine Science and Technology, Qingdao 266003, China

**Keywords:** Trochoidea, species delimitation, ABGD, ASAP, GMYC, PTP

## Abstract

In the context of diminishing global biodiversity, the validity and practicality of species delimitation methods for the identification of many neglected and undescribed biodiverse species have been paid increasing attention. DNA sequence-based species delimitation methods are mainly classified into two categories, namely, distance-based and tree-based methods, and have been widely adopted in many studies. In the present study, we performed three distance-based (ad hoc threshold, ABGD, and ASAP) and four tree-based (sGMYC, mGMYC, PTP, and mPTP) analyses based on Trochoidea COI data and analyzed the discordance between them. Moreover, we also observed the performance of these methods at different taxonomic ranks (the genus, subfamily, and family ranks). The results suggested that the distance-based approach is generally superior to the tree-based approach, with the ASAP method being the most efficient. In terms of phylogenetic methods, the single threshold version performed better than the multiple threshold version of GMYC, and PTP showed higher efficiency than mPTP in delimiting species. Additionally, GMYC was found to be significantly influenced by taxonomic rank, showing poorer efficiency in datasets at the genus level than at higher levels. Finally, our results highlighted that cryptic diversity within Trochoidea (Mollusca: Vetigastropoda) might be underestimated, which provides quantitative evidence for excavating the cryptic lineages of these species.

## 1. Introduction

In the context of the gradual reduction in and loss of global biodiversity [1,2,3,4,5,6,7], it is necessary to use taxonomic knowledge to quantify and delimit species diversity. An increasing number of researchers support the use of molecular information to assist taxonomy, which can not only expedite the proper identification and delimitation of species, but can also provide effective means for the discovery and documentation of new taxa [8,9,10]. Methods for species delimitation relying on molecular information can generally be divided into single- or multi-locus methods [11]. A variety of approaches using single-gene data have become important for studies on hundreds or thousands of species [12,13]. Despite multi-locus studies having significant advantages in simultaneously considering several loci when delimiting species, single-locus methods still predominate in the literature [14,15,16]. Moreover, single-gene data also have much lower costs and have relatively smaller computational requirements. The use of single-locus data, rather than multi-locus data, as the primary evidence for identifying species, has enabled the rapid and effective generation of species hypotheses. As a consequence, the single-locus species delimitation method has become an extremely effective molecular tool when applied to integrative taxonomy, which is a multisource approach to exploring biodiversity [17]. Additionally, the single-gene methods are likely to continue to be used for many years. However, even if the single-locus methods have some advantages, the species hypotheses proposed by this approach are generally less robust and need to be complemented by other approaches.

Single-locus methods can generally be classified into two types: distances-based and tree-based methods [18,19,20]. Ever since the concept of a DNA barcode was proposed [21], a vast number of studies have aimed to verify its validity and practicality by comparing the knowledge of species boundaries obtained with morphospecies [22,23]. One of the first methods proposed for delimiting species from the cytochrome c oxidase subunit I (COI) barcode dataset was based on the pairwise nucleotide genetic distances of sequences [24]. This approach tries to find a “barcoding gap”, which is the assumption that the amount of genetic variation within species is smaller than the amount of variation between species [25]. Although efforts have been made to establish a fixed threshold to distinguish between intra- and inter-species divergence (e.g., 3% of divergence [26] or the 10 × rule [21]), it cannot apply to all groups of organisms when taxa with different evolutionary histories are analyzed together [25,27]. Therefore, the application of a group-specific ad hoc threshold should lead to more accurate delimitations. From this point of view, the automatic barcode gap discovery (ABGD) method was proposed [19] and has been widely used in several studies [13,28,29]. The method seeks to search for a “barcode gap” in the distribution of pairwise sequence divergences and partitioning of the data is repeated until no further splitting occurs. The hierarchical clustering algorithm mainly uses a series of prior intraspecific divergences to infer the unilateral confidence interval limit for intraspecific divergence. The barcode gap is used as a threshold to delimit molecular operational taxonomic units (MOTUs) [30]; the confidence limit is deduced, and the barcode gap is detected recursively until the MOTUs cannot be divided [19]. Subsequently, the updated implementation of the ABGD, named “assemble species by automatic partitioning” (ASAP), was described by Puillandre et al. [31], which delimits species without prior biological insight into intraspecific diversity and provides an asap-score system used to rank the alternative partitioning schemes.

In contrast to the previous methods, the tree-based method uses tree topology instead of relying on the clustering of haplotype sequences [32]. This method is mainly classified into two types: the generalized mixed Yule coalescence (GMYC) [33] and Poisson tree processes (PTP) [34]. In recent years, the GMYC model has proven to be a practical tool by which to delimit species from single-locus information in datasets due to its robustness and accuracy [12,35,36,37]. This method takes an ultra-metric tree as an input and attempts to detect the transition of branching patterns from interspecific branches to intraspecific branches. The principle of this strategy is to estimate the rates of branching events in order to infer two categories, i.e., speciation and coalescence within species. GMYC methods were first implemented under a maximum likelihood framework, including a single-threshold [33] and a multi-threshold version [38], and then expanded to a Bayesian framework [28]. In contrast to GMYC, the PTP model is designed to use the number of substitutions rather than time to estimate branching processes and, thus, requires an ML tree as input, which results in it being faster and easier to run than GMYC [34,39]. In principle, the number of substitutions is expected to be significantly higher between species than within species. For PTP, assuming that each substitution has a small probability of generating a speciation, and that the number of substitutions in a population of a certain size is large, the process follows a Poisson distribution in continuous time [34]. The original PTP model was then expanded to its Bayesian implementation (bPTP), which adds Bayesian support (BS) values to the input tree. Recently, an improved method, named the multi-rate PTP (mPTP), was proposed by Kapli et al. [40], which fits a distinct exponential distribution to each delimited species to make explicit differences in the evolutionary process or sampling intensity.

The performance of single-locus species delimitation methods has been tested in chordates, arthropods, molluscs, and other biological groups [13,27,41,42,43,44]. Although many studies have primarily aimed to examine species diversity, these have effectively compared the results obtained from different species delimitation approaches. For example, Padula et al. [45] showed that the GMYC approach was inclined to split species compared with ABGD and PTP; Knutson and Gosliner [46] showed that the conservative results obtained by ABGD and PTP were mostly concordant, even if there were some differences. Trochoidea (Gastropoda: Vetigastropoda), which is the largest and most biodiverse superfamily within Vetigastropoda [24,47], consists of more than 2000 living species that are grouped into about 237 recognized genera in WORMS (https://www.marinespecies.org/, accessed on 20 August 2022). It is an excellent model for comparative analyses of the efficiency of MOTU-delimitation algorithms in recovering species boundaries because a large number of molecular sequences have been published and used to study the complex phylogenetic relationships based on single-loci or genomic data [48,49,50]. Given that more empirical studies should be used to compare and contrast the results between species delimitation methods and nominal species, in this study, the primary goal was to compare the efficiency of single-locus species delimitation methods within Trochoidea. Moreover, we were interested in the performance of species delimitation methods at different taxonomic levels. Finally, we highlight that the consistency of results obtained from multiple tests should be considered before drawing conclusions of the cryptic diversity of species.

## 2. Materials and Methods

### 2.1. DNA Extraction, PCR Amplification, and Sequencing

The total genomic DNA was extracted from 5 to 10 mg of foot tissue using the TIANamp Marine Animals DNA Kit (TIANGEN Biotech Beijing Co., Ltd., Beijing, China), following the manufacturer’s protocols. A partial region of the COI gene was amplified using a universal primer pair: LCO1490 (5′-GGTCAACAAATCATAAAGATATTGG-3′) and HCO2198 (5′-TAAACTTCAGGGTGACCAAAAAATCA-3′) [51]. The polymerase chain reaction (PCR) was carried out in a final volume of 25 µL and contained 22µL of GoldenStar^®^ T6 Super PCR Mix (1.1×) (Tsingke Biotechnology Co., Ltd., Beijing, China), 1 µL of each primer, and 1µL of template DNA. Thermocycler conditions were set with initial denaturation at 94 °C for 3 min, followed by 35 cycles of denaturation at 94 °C for 30 s, annealing at 48 °C for 30 s, elongation at 72 °C for 1 min, and final elongation at 72 °C for 10 min. PCR products were confirmed by electrophoresis in 1.5% agarose gel and the qualified products were sent to Sangon Biotech (BGI TECH SOLUTIONS Co., Ltd., Beijing, China) for purification and bidirectional sequencing.

### 2.2. Sequence Analysis and Dataset Composition

In the present study, we combined 1248 previously published COI sequences of Trochoidea in GenBank (Table S1) with our 108 new COI sequences (Table S2). Chromatograms were examined and single consensus sequences for each individual were generated using Seqman software v.7.1 (DNASTAR, Inc., Madison, WI, USA) [52]. In terms of sequence selection, ambiguous taxonomic labels (e.g., “*Arene* sp.”) were excluded because they are meaningless when comparing the results of species delimitation methods. Additionally, we only analyzed sequences that were at least 500 bp in length in order to meet the minimum length of sequences required for the DNA barcode [20]. A series of 1356 sequences was assigned to 40 datasets according to the taxonomic levels, resulting in 12 families, 7 subfamilies, and 21 genus datasets (Table 1). Then, multiple sequence alignment was implemented using MAFFT v.7.4 [53], and the sequences were adjusted and trimmed using TrimALv1.4 [54] with default parameters in a Linux environment. For each dataset, identical haplotypes were removed using ALTER [55] because the GMYC model can only handle dichotomous branches [12,56].

**Figure 1:**
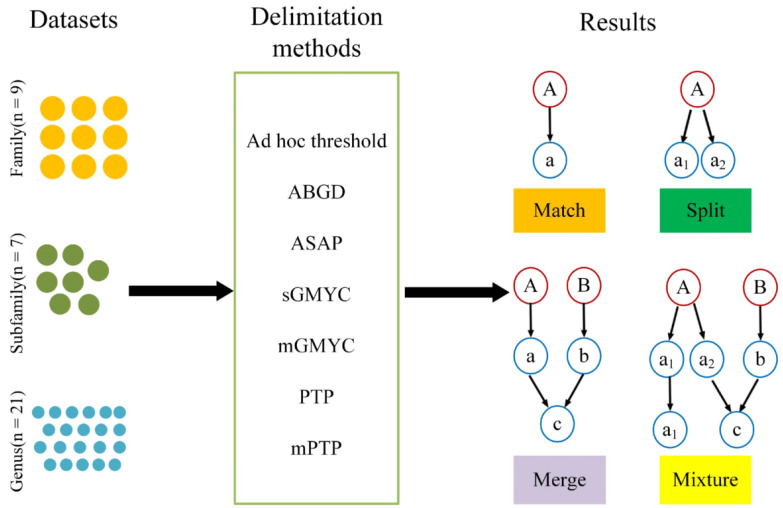
Datasets analyzed, species delimitation methods, and the categorization of results in the study. “n=” refers to the total number of datasets. The red circle represents nominal species and the blue circle represents the MOTUs.

The existence of a “barcode gap” for each dataset was evaluated using the “Spider” package [57] in R version 3.6 [58] by calculating the difference between the minimum interspecific distance and the maximum intraspecific distance. Our results showed that a zero difference existed in the Angariidae dataset. ABGD cannot propose a primary partition and is therefore not suitable for species delimitation when no barcode gap exists in a dataset [19]. Since the Areneidae dataset was composed of only one sequence, it could not be applied for species delimitation. Moreover, tree-based methods were used on the phylogenetic trees by excluding the Skeneidae dataset with only one sequence per species. Therefore, in our study, all the analyses were performed on 37 haplotype datasets, including 9 families, 7 subfamilies, and 21 genus datasets (Figure 1). The datasets varied greatly in terms of species and COI sequence numbers, which ranged from the family dataset, composed of 130 species and 411 sequences, to the genus dataset, containing 3 species and 4 sequences (Table 1). The genus datasets included 8.3 species on average; the maximum number of species contained was 28, and the minimum was 3 species. The number of species in the subfamily datasets ranged from 6 to 80, with a mean of 28.9 species. The family datasets contained a mean of 32 species, with the number ranging from 2 to 130 species.

### 2.3. Phylogenetic Analysis

Prior to the species delimitation analyses, we selected the best-fit model of nucleotide substitution for the dataset using BIC in MEGA X [59] based on the Bayesian information criterion. Two phylogenetic trees were obtained for each dataset as follows: a maximum likelihood (ML) tree calculated in RAxML-HPC2 v8.2 [60] and an ultra-metric gene tree in BEAST v.2.6 [61]. ML trees were obtained in the Cyber Infrastructure for Phylogenetic Research (CIPRES) portal v.3.1 [62], under the GTR+γ model of evolution, using 200 heuristic independent runs and a bootstrap resampling of 1000 replicates. With respect to the ultra-metric tree for the GMYC analyses, under a strict clock, yule tree prior, and all other priors were left at the default values. A Markov chain length ranging from 10 × 10^6^ to 80 × 10^7^ generations, depending on the estimated sample size of each parameter of the model, and a burn-in of 0.1, trace logs were visualized in Tracer version 1.7 [63] to assess convergence and adequate posterior sampling (ESS > 200). A maximum clade credibility (MCC) tree was created in TreeAnnotator v.2.6 [61] using mean heights for annotation. Additionally, FigTree 1.4.4 [64] was used to visualize and edit the trees.

### 2.4. Molecular Species Delimitation Analyses

In this study, three distance-based and four tree-based methods were used for the production of species hypotheses, as follows: (1) the ad hoc estimated threshold on each dataset [27]; (2) ABGD [19]; (3) ASAP [31]; (4) the single-threshold GMYC (sGMYC) model [33] and the multi-threshold GMYC (mGMYC) model [38]; and (5) the PTP model [34] and the multi-rate PTP (mPTP) model [40].

In order to assess the efficiency of the different species delimitation methods, we adopted the strategy used by Magoga et al. [27] to compare the results of a variety of species delimitation approaches. The efficiency of each method in delimiting species was evaluated by examining the correspondence between molecular delimited units and nominal species for each dataset. The efficiency of each method in recovering boundaries was assessed according to the following categories: (1) match: all sequences belonging to a species are assigned to an MOTU that contains no other members; (2) split: the sequences of a species are divided into two or more MOTUs; (3) merge: all the sequences of one species are placed in a single MOTU along with all the sequences of another species; (4) mixture: some sequences of a species are split, while others are merged.

As the threshold value of the barcode gap is variable between groups [18], the R function “localMinima” of the “Spider” package was used to compute the minimum value in the density of the nucleotide distances as the most likely threshold for species delimitation in the dataset. Then, we took advantage of the R function “tclust” of the “Spider” package to cluster sequences at the previously identified ad hoc threshold value. Our analyses were processed in the command-line of the ABGD using the Kimura two-parameter (K2P) substitution model [65] and a gap width (X) of 1.5; when a gap was not found using this value, a width of 1.0 or 0.5 was set, the prior maximum value of intraspecific divergence ranging from 0.001 to 0.1 and other parameters were left as default. For ABGD, only the primary partitions were considered since they were typically stable on a wider range of prior values. ASAP analyses were run under the Linux environment; K2P was selected as the nucleotide substitution model and the remaining parameters were left as default. This method mainly overcomes the limitations of ABGD in two respects. On the one hand, it no longer requires users to provide a prior limit to intraspecific diversity (p). On the other hand, it provides users with an ASAP score for each partition, which allows the users to choose the “best” partition of species delimitation [31]. For ASAP, we selected the partition that was closest to the delimitation of nominal species among the ten partitions. For GMYC delimitation, the sGMYC and mGMYC methods were both conducted in R, using the “Splits” (Species Limits by Threshold Statistics) package [66]. The main function of “Splits” was to test the fit of a GMYC model versus a null model of coalescence. For the PTP and mPTP methods, we performed ML and MCMC analyses using an ML tree in the command line, and both utilized the –multi option to incorporate differences in the rates of coalescence among species. For each dataset, a sequence was selected as an outgroup to root the phylogenetic tree based on current systematic knowledge and it was removed before running the delimitation analysis. We performed 10 different runs with the following settings: mcmc run of 10 million generations, sample every 5000, and the first million generations were discarded as burn-ins. Convergence was assessed by observing the output plot of generation vs. log-likelihood (created using the “-mcmc_log” command). The congruency of the independent runs was assessed according to the average standard deviation of the delimitation support values (ASDDSV), which approached zero as the multiple MCMC runs converged on the same delimitation distribution. In addition, we assessed the confidence of the ML delimitation according to the average support values (ASV) over the 10 runs.

## 3. Results

**Figure 2:**
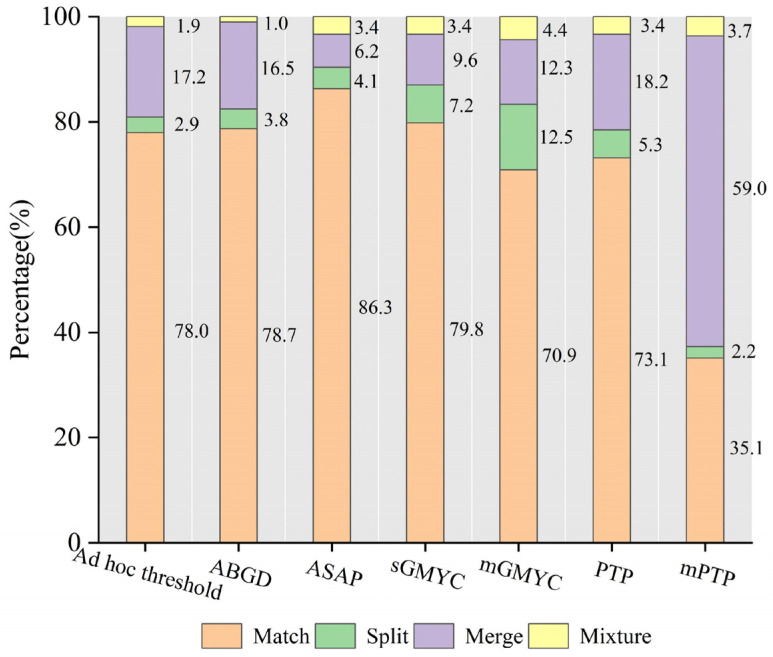
Efficiency of the species delimitation methods applied to the 37 analyzed datasets. The y-axis is the mean percentage of matches (orange), splits (green), merges (purple), and mixtures (yellow) observed for each approach. The primary partitions of ABGD and the partition closest to the delimitation of nominal species in ASAP were considered as the final results.

In this study, the number of matches obtained from each analysis represents the efficiency of the species delimitation methods. Our results showed that molecular delimitation using ASAP resulted in the highest percentage of matches (86.3% on average) between the estimated MOTUs and the nominal species. A similar percentage of matches was obtained using the ad hoc nucleotide distance threshold (78.0% on average, Table S3) and ABGD (78.7% on average), which were moderately lower than when using sGMYC (79.3% on average) (Figure 2, Table S4). Regarding the analysis of the results of sGMYC and mGMYC (79.8% and 70.9% on average, respectively), these both demonstrated over-splitting of species compared with the other approaches. For the PTP analyses, the ASDDSV was ≤0.01 for all the datasets, suggesting the better convergence of the 10 independent MCMC runs. Additionally, for the majority of the datasets (32 out of 37 datasets), ASV exceeded 50%, suggesting that the ML analysis was well-supported by our data. For the mPTP analyses, the ASDDSV was ≤0.01 in 36 out of 37 datasets; for the six datasets with ASV values under 50%, these should be considered as having no significance in the results. In comparison with the matching number of PTPs (73.1% on average), mPTP showed a lower proportion of matches (35.1% on average) and a higher percentage of merges (59.0% on average) (Figure 2 and Table S4).

There were also considerable similarities in the performance of GMYC and PTP regarding phylogenetic-coalescent methods. Our results showed that a lower number of matches was obtained with the multi-threshold version than the single-threshold version of GMYC and PTP. Additionally, the GMYC method tended to split species, and molecular delimitation using sGMYC and mGMYC resulted in a higher percentage of splits (7.2% and 12.5% on average, respectively, Figure 2).

**Figure 3:**
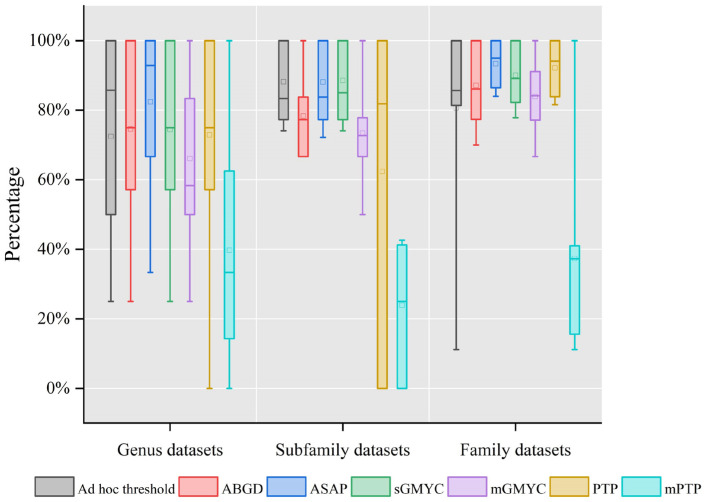
The y-axis represents match percentage observed for each method in three taxonomic levels (genus, subfamily, and family). The square in the bar represents the average of the data; the horizontal line in the bar represents the median of the data; the lines parallel to the columns are the lines between the maximum and minimum values in each set of data.

Taxonomic rank is one of the factors affecting the efficiency of species delimitation methods [27]. In our study, we tested these methods on several replicates of the same dataset, aggregated on the base of growing taxonomic ranks, for example, by grouping all the species of one genus, all the genera of a subfamily, and all the subfamilies of a family. In genus-level datasets, we distinctly observed the better efficiency of the distance-based methods compared with the tree-based methods (Figure 3). In addition, the species delimitation efficiency of ASAP was the highest in the genus- and family-level datasets, while it was slightly inferior to sGMYC in the subfamily datasets. At the subfamily level, using the ad hoc threshold, ASAP and sGMYC showed almost equal efficiency in recovering species boundaries. Moreover, it can be easily observed that the data were mostly concentrated on a smaller scale and analyzed at the level of the family (Figure 3). In accordance with the conclusion of Magoga et al. [27], GMYC was significantly affected by taxonomic rank and had poor efficiency when used on our genus-level datasets compared with other levels (Figure 3).

## 4. Discussion

### 4.1. Species Delimitation Method Efficiency

Our results showed that different methods do produce different delimitation scenarios based on single-locus data, which is congruent with the findings of previous studies [32,67]. Considering all the approaches adopted in this study, the distance-based method analyses generally outperformed the coalescent-derived method analyses, with the highest efficiency being from ASAP, followed by sGMYC and ABGD. The ASAP method showed the 10 best partitions with species delimitation. Although lower ASAP scores indicated better partitions for species delimitation [31], only 78% of the analyzed datasets achieved a high percentage of matches in one of the two best ASAP-score partitions. This reminds us that we should not only consider the two optimal partitions of ASAP, but also that it is better to combine the partitions with biological knowledge or other characteristics to delimit species in an integrative taxonomy framework. In addition, only 64% of the analyzed datasets were identical to the partition selected as the “best” from the ABGD output. Certainly, it should be taken into consideration that these methods based on pairwise distance, to a large extent depend on the choices of the users, namely, the use of the ad hoc threshold, the initial and recursive partition of ABGD, or the partition selection of ASAP.

It is well-known that GMYC is prone to over-splitting species [45,56], especially the multi-threshold version of GMYC [68,69]. The mGMYC method is very sensitive to deep coalescent events since it allows many temporal thresholds for speciation, resulting in the overestimation of the number of species [70]. For the PTP model, species were significantly prone to being over-lumped by mPTP in our study (Figure 2), which was contrary to what was observed to occur in the splitting of haplotype-rich species [13,31]. There are many factors contributing to this result, such as the ratio of population sizes to species divergence times, effective population size, variation among species, uneven sampling, and gene flow [32,70,71]. A high proportion of singletons may also hinder the performance of these two methods in species delimitation [12,34]. Some issues lead to incongruence in tree-based methods for species delimitation. For instance, the zero-length terminal branches of trees were considered as a relevant point for the correct identification of the transition point between coalescent and speciation processes [39,42]. Moreover, the process of the intraspecific diversification can also result in phylogenetic arrangements with long branches, which might produce a deviation for estimating species diversity [70].

However, in our study, all the results obtained were only based on a single locus. Therefore, in future studies, we will consider repeating the same analyses using other loci to determine whether the conclusions remain unaltered. Additionally, for given datasets, the incongruence in the results across methods also confirmed the individual shortcomings of one or more of the methods used to delimit species. Therefore, a more appropriate means of delimiting species is to analyze these data using a wide range of methods.

### 4.2. Cryptic Diversity

**Figure 4:**
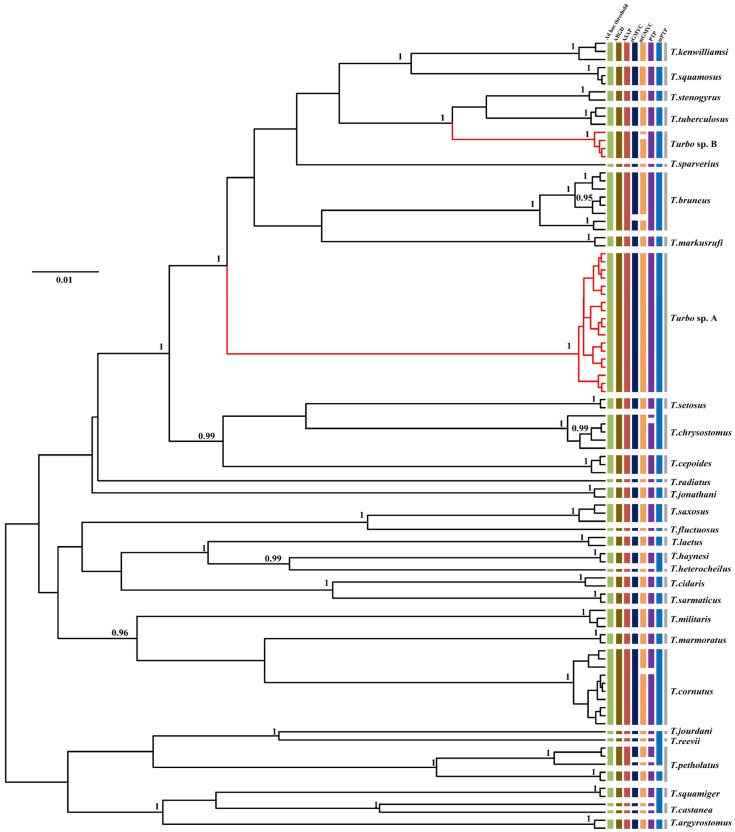
Comparison of species delimitation results of *Turbo* based on analysis of COI sequences of 98 individuals from 28 different nominal species and two unclassified clades (*Turbo* sp. A and *Turbo* sp. B) highlighted in red, with lineage assignments from the four tree-based (PTP, mPTP, sGMYC, mGMYC) and three distance-based (ad hoc threshold, ABGD, ASAP) methods. Each colored bar represents a species delimited by each method tested and gray bars represent nominal species. Gene tree is from a BEAST analysis and MCC tree is shown. Node values represent Bayesian posterior probabilities (≥0.95) for major clades.

**Figure 5:**
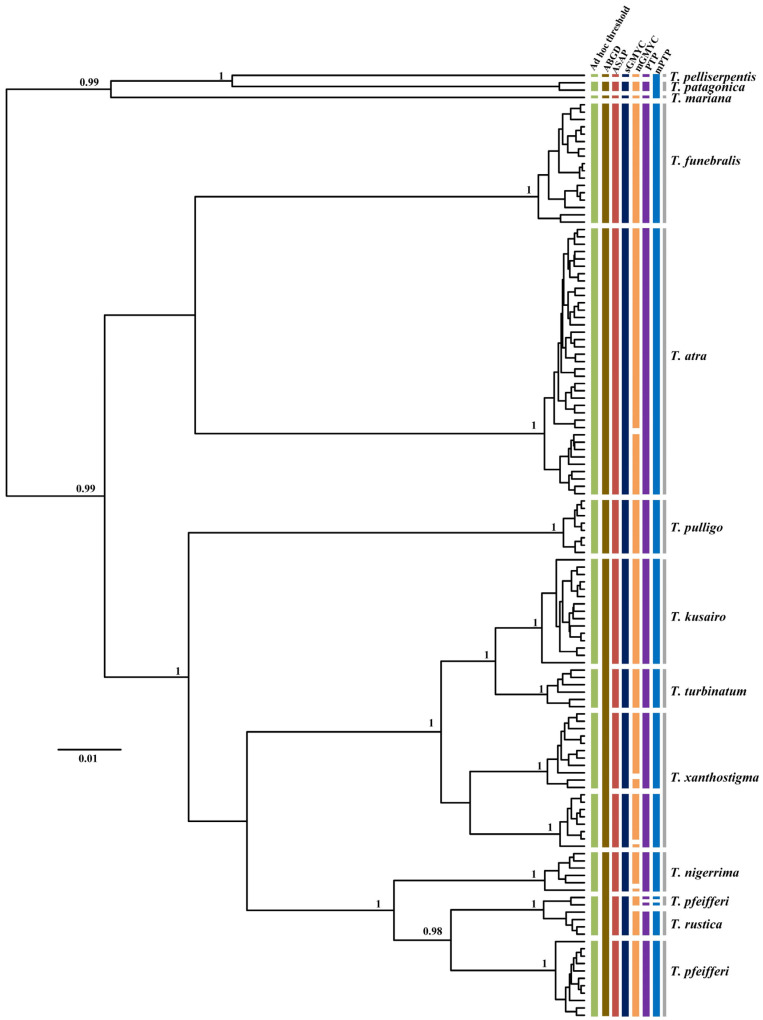
Comparison of species delimitation results of *Tegula* based on analysis of COI sequences of 149 individuals from 12 different nominal species, with lineage assignments from the four tree-based (PTP, mPTP, sGMYC, mGMYC) and three distance-based (ad hoc threshold, ABGD, ASAP) methods. Each colored bar represents a species delimited by each method tested and gray bars represent nominal species. Gene tree is from a BEAST analysis and MCC tree is shown. Node values represent Bayesian posterior probabilities (≥0.95) for major clades.

**Figure 6:**
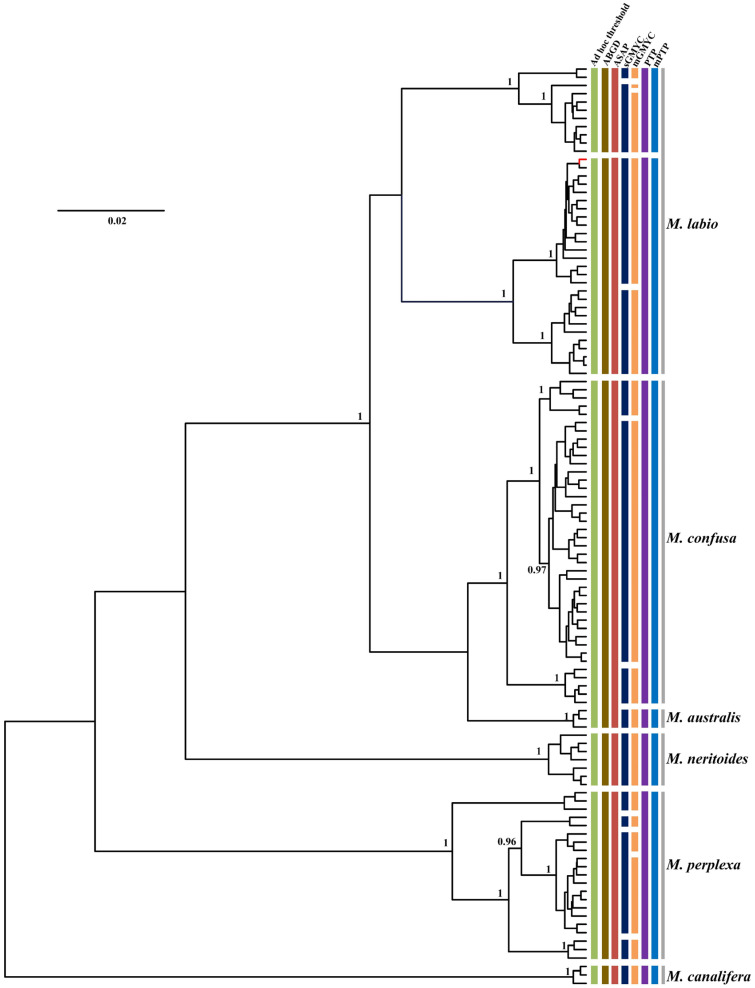
Comparison of species delimitation results of *Monodonta* based on analysis of COI sequences of 112 individuals from six different nominal species, with lineage assignments from the four tree-based (PTP, mPTP, sGMYC, mGMYC) and three distance-based (ad hoc threshold, ABGD, ASAP) methods. Each colored bar represents a species delimited by each method tested and gray bars represent nominal species. Gene tree is from a BEAST analysis and MCC tree is shown. Node values represent Bayesian posterior probabilities (≥0.95) for major clades. The red branch represents one individual of *M. confusa*.

**Figure 7:**
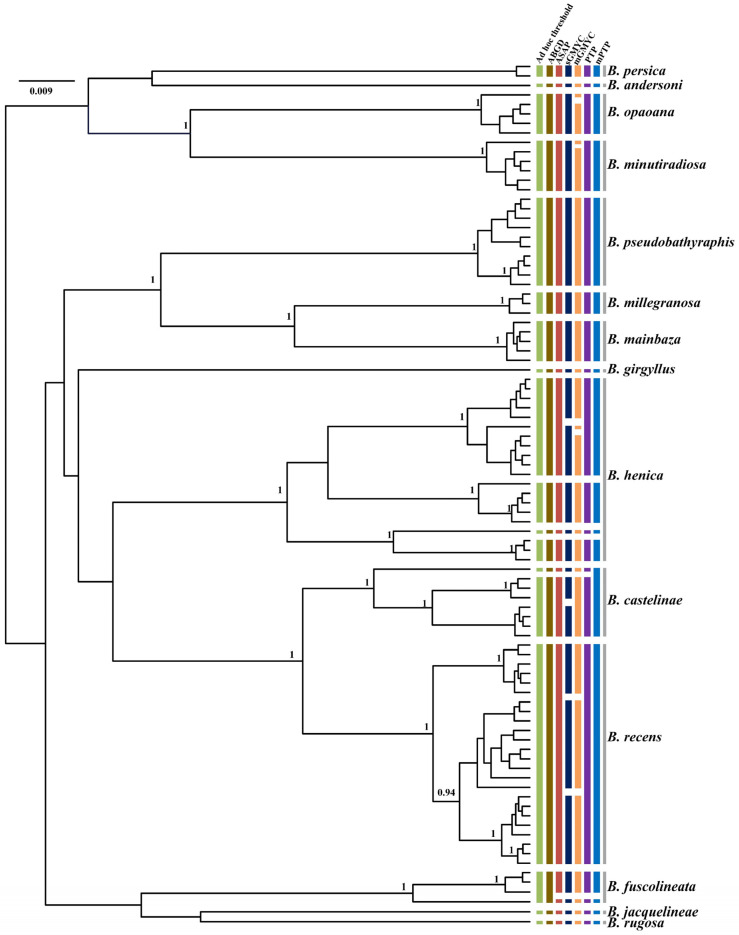
Comparison of species delimitation results of *Bolma* based on analysis of COI sequences of 91 individuals from 14 different nominal species, with lineage assignments from the four tree-based (PTP, mPTP, sGMYC, mGMYC) and three distance-based (ad hoc threshold, ABGD, ASAP) methods. Each colored bar represents a species delimited by each method tested and gray bars represent nominal species. Gene tree is from a BEAST analysis and MCC tree is shown. Node values represent Bayesian posterior probabilities (≥0.95) for major clades.

Incongruence across one or more of the methods used for delimiting species is likely to be useful evidence in the detection of cryptic lineages. In this study, by comparing the inconsistence between seven species delimitation approaches, we observed four genera (*Turbo*, *Tegula*, *Monodonta,* and *Bolma*) whose phylogenetic relationships remained ambiguous. Our results showed that several nominal species were included in a single MOTU, while a few recognized species were inferred to represent two or more MOTUs. In *Turbo*, these methods congruently inferred multiple isolated species-level lineages within *Turbo castanea*, while mGMYC additionally inferred multiple lineages within *Turbo chrysostomus* and *Turbo corutus* (Figure 4). Conversely, PTP and mPTP inferred *Turbo petholatus* as the same lineage, but as distinct lineages in the other five methods. Moreover, two morphologically similar, but genetically polyphyletic, groups were identified within *Turbo* (*Turbo* sp. A and *Turbo* sp. B). All the samples of *Turbo* sp. A were collected from Hainan province and all the specimens of *Turbo* sp. B were collected from Guangxi province (Table S2). Moreover, the genus *Tegula* was found to have some unclear taxonomic relationships on account of its higher intraspecific morphological variation. *Tegula pfeifferi* was observed to have two distinctive morphotypes based on its shell surface (smooth and ribbed) and *Tegula xanthostigma* had two shell colors (black and light brown) [49,72]. According to the recovered species boundaries using the ad hoc threshold with ASAP and sGMYC (Figure 5), two *T. pfeifferi* individuals were of the ribbed type and all the samples of *Tegula rustica* were incorporated into one MOTU. However, other *T. pfeifferi* individuals with either a ribbed or smooth surface were assigned to another MOTU. Therefore, the phylogenetic relationship between *T. rustica* and *T. pfeifferi* needs to be further studied [49]. Additionally, for *T. xanthostigma*, individuals with different shell colors were divided into two MOTUs by all the methods, excluding ABGD and mGMYC (Figure 5), which suggested that the trait shell color morphotypes were genetically distinguishable. The conclusions above are consistent with the phenomenon described by Yamazaki et al. [49]. Moreover, in *Monodonta*, within three species, named *Monodonta labio*, *Monodonta australis,* and *Monodonta confusa*, respectively, the number of MOTUs inferred by unilocus species delimitation methods was mismatched with the number of morphologic entities. All the approaches, except sGMYC and mGMYC, divided the individuals of *M. labio* into two lineages. One lineage not only included *M. labio*, but also contained one individual of *M. confusa*. *M. australis* and the remaining haplotypes of *M. confusa* were lumped by the distance-based methods, but the tree-based approaches could clearly distinguish the two species (Figure 6). The remaining haplotypes of *M. confusa* were assigned to three MOTUs by the GMYC method, which further demonstrated that the GMYC method is characterized by the over-splitting of species compared with the other approaches. In the genus *Bolma*, shell morphology was considered of poor significance in species identification due to its higher adaptation [73]. Some species belonging to *Bolma* showed high levels of intraspecific genetic differentiation, such as *Bolma henica*, *Bolma castelinae*, and *Bolma fuscolineata* (Figure 7). Although the discordances between nominal species and genetic entities based on COI or genomic data were studied within Trochoidea [49,73,74], we used more single-locus species delimitation methods to further confirm this phenomenon and provided evidence for probing the cryptic lineages of these species.

There are many factors resulting in the splitting of species. For example, the splitting of the oceans leads to high levels of genetic differentiation in *M. labio* [74]. In addition, different preferred habitats might trigger genetic divergence among populations, such as *T. xanthostigma* [49]. Certainly, the situation of lumping or mixing species also exists, which might be explained by incomplete lineage sorting in ancient lineages or the emergence of hybridization due to incomplete reproductive isolation [49,75]. For instance, *M. confusa* and *M. labio* were genetically distinguished, but one individual of *M. confusa* was gathered with *M. labio*, which may be due to hybridization having occurred between *M. confusa* and *M. labio* [74] (Figure 6). Furthermore, for shelled molluscs, the problem of shell polymorphism and conservatism is a huge challenge for species identification [73]; for instance, the high level of intraspecific shell polymorphism in *Bolma* and the different shell sculptures and colors existing in *Tegula*. In these cases, using the molecular data rather than morphological characters as primary evidence of species identification can objectively produce species hypotheses. Therefore, further taxonomic investigation into these lineages is essential. Finally, we suggest that additional molecular analyses using multi-locus data or SNPs obtained by next-generation sequencing are necessary for further studies of these ambiguous taxonomic problems.

## 5. Conclusions

In this study, we took advantage of multiple datasets composed of COI data to test a series of unilocus species delimitation methods based on pairwise genetic distance and phylogenetic trees. By examining the number of matches between nominal species and inferred MOTUs using these approaches, we found that the efficiency of the distance-based and tree-based approaches had certain variance in recovering species boundaries. The results suggested that the distance-based approach was generally superior to the tree-based approach, with the ASAP method being the most efficient. Additionally, the single-threshold version of GMYC performed better than the multi-threshold version in general. The PTP method showed higher efficiency than mPTP in delimiting species. Moreover, GMYC was found to be significantly influenced by the taxonomic rank, showing poorer efficiency in datasets at the genus level than at higher levels. In addition, our results further confirmed that the different species delimitation methods produced different results, which strongly supports the necessity of using a combination of methods before reaching a final species hypothesis. Moreover, we emphasize that there is a need to explore previously unrecognized species diversity in order to lay a foundation for taxonomic and systematic investigation. However, other factors affecting species delimitation should also be taken into consideration when drawing conclusions regarding the efficiency of these methods. Considering that we compared the efficiency between these methods based only on one locus, in the future, we will use other loci to repeat the same analyses to confirm whether the conclusions here remain unchanged.

## Figures and Tables

**Table 1:** The number of species and sequences contained in 40 datasets.

Datasets	Taxonomic Rank	Number of Nominal Species	Number of Sequences	Number of Haplotypes
*Astralium*	Genus	12	21	21
*Austrocochlea*	Genus	7	58	32
*Bolma*	Genus	14	166	91
*Calliostoma*	Genus	11	58	27
*Cantharidus*	Genus	7	23	14
*Clanculus*	Genus	6	8	7
*Diloma*	Genus	10	18	17
*Gibbula*	Genus	6	44	40
*Homalopoma*	Genus	6	19	10
*Jujubinus*	Genus	4	5	5
*Lirularia*	Genus	3	6	6
*Lunella*	Genus	10	80	42
*Micrelenchus*	Genus	6	14	12
*Monodonta*	Genus	6	198	112
*Phorcus*	Genus	8	93	61
*Steromphala*	Genus	7	54	37
*Tectus*	Genus	3	5	4
*Tegula*	Genus	12	149	107
*Trochus*	Genus	3	19	7
*Turbo*	Genus	28	76	59
*Umbonium*	Genus	5	12	10
Cantharidinae	Subfamily	54	254	193
Fossarininae	Subfamily	6	9	7
Monodontinae	Subfamily	22	274	160
Stomatellinae	Subfamily	6	11	9
Trochinae	Subfamily	16	37	23
Turbininae	Subfamily	80	375	226
Umboniinae	Subfamily	18	32	25
Angariidae	Family	2	3	2
Areneidae	Family	1	1	1
Calliostomatidae	Family	17	64	33
Colloniidae	Family	9	22	13
Liotiidae	Family	2	4	3
Margaritidae	Family	9	19	12
Phasianellidae	Family	5	9	9
Skeneidae	Family	7	7	7
Solariellidae	Family	15	45	39
Tegulidae	Family	20	159	116
Trochidae	Family	130	624	411
Turbinidae	Family	81	377	228

## Data Availability

The original sequencing data have been submitted to GenBank (accession number ON908702-ON908706; ON908817-ON908836; ON908877-ON908879; ON908885-ON908888; ON908946-ON908951; OP457006-OP457075).

## Supplementary Materials

The following supporting information can be downloaded at: https://www.mdpi.com/article/10.3390/genes13122273/s1, Table S1: GenBank accession number of all COI sequences analyzed in our study; Table S2: The sample localities and GenBank accession number of sequences; Table S3: Nucleotide distance threshold values for species delimitation ad hoc estimated for each dataset; Table S4: Molecular species delimitation analyses results.
